# Ebola and the need for restructuring pharmaceutical incentives

**DOI:** 10.7189/jogh.05.010303

**Published:** 2015-06

**Authors:** Abraar Karan, Thomas Pogge

**Affiliations:** 1 David Geffen School of Medicine at UCLA, Los Angeles, CA, USA; 2 Yale University, Department of Philosophy, New Haven, CT, USA

The Ebola outbreak in West Africa has claimed the lives of over 9000 people largely due to a combination of poor health care infrastructure in affected countries, traditional beliefs and cultural practices, including the consumption of bushmeat and certain burial rituals that have amplified transmission, and the lack of therapeutic interventions such as medications and vaccinations [1,2]. Ebola virus was discovered in 1976, and since then there have been over 30 outbreaks, the majority occurring in Sub-Saharan Africa, yet development of medications has been negligible [3]. Moreover, while the current epidemic has spurred a new race to develop Ebola vaccines and treatment regimens, the current patent system makes it unlikely that people in the most afflicted nations will have access to such vaccines or medications when they are brought to market without the assistance of development aid initiatives from the United Nations (UN), World Health Organization, the GAVI Alliance and other multinational global entities.

While there have been just a handful of deaths outside of Africa, the vast majority of fatalities from Ebola virus have been in low-income African countries. This is largely because wealthy nations have been able to mount strong public health responses through providing effective medical care to stabilize patients, enforcing strict isolation protocols to prevent further transmission, and accessing experimental therapies for use in their populations, including ZMapp and TKM-Ebola [4]. Several other drugs and vaccines are also under rapid development, most notably ChAd3, which was recently highlighted in the *New England Journal of Medicine* as having immunogenicity in humans [5]. According to a February 2015 press release from the UN and WHO, large-scale research trials have now begun in Liberia, with Sierra Leone and Guinea to follow soon [6]. But when these drugs are fully approved for international distribution, will they be affordable for all? Given the current global drug-patenting paradigm with its 20-year delay on generic competition, patent holders can set drug prices as high as they please, effectively making their drugs inaccessible to poor populations.

Moreover, with a limited supply of Ebola medications even in the near future, wealthy nations will likely stockpile the drugs and vaccines as was done with Tamiflu in 2009 [7], preventing poorer nations from accessing therapy to treat those who are currently infected. There is no financial or political mechanism to ensure that drugs and vaccines are available and affordable for the people of Guinea, Liberia, Sierra Leone and other poor nations at high risk of Ebola epidemics. As of December 2014, the GAVI Alliance has made a commitment of US$ 300 million to purchase Ebola vaccines for those in affected countries, but this is only an *ad hoc* solution as opposed to a fundamental restructuring of the system [8].

**Figure:**
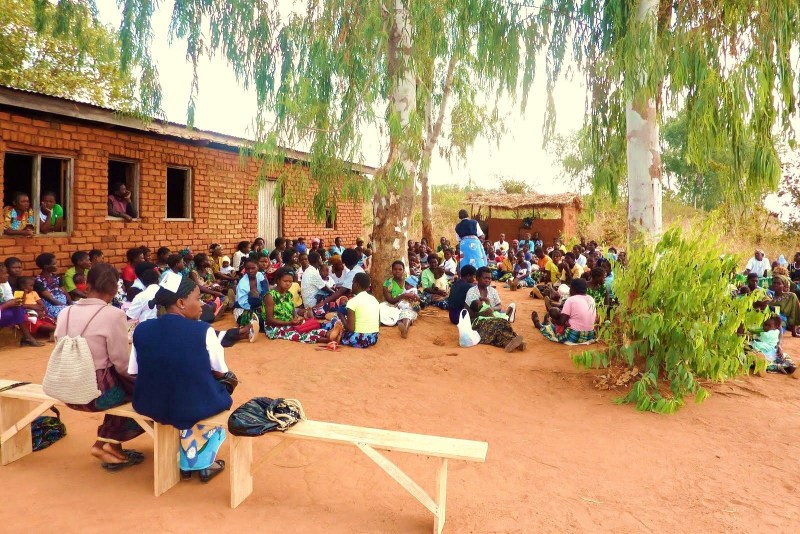
Photo: Courtesy of Alasdair Campbell, personal collection

The affordable provision of treatment for people in West Africa is not only an ethical imperative, but also the best strategy to keep Ebola from spreading to other continents on a larger scale. Ultimately, the international community must intervene to ensure that future Ebola medications are sold at a tiered price to developing countries that are most heavily afflicted. But it remains unclear if this can or will happen.

While making Ebola medications accessible to all will be the challenge going forward, we should also ask why no therapy for this high-fatality virus was brought to market since its discovery 40 years ago. The reason lies in the way our pharmaceutical innovation system is structured. Four years ago, scientists at the National Institute of Allergy and Infectious Disease developed an Ebola vaccine that was able to prevent animal transmission, but no pharmaceutical company was interested in taking it to trial in human subjects [9]. While there are programmes, such as the USAID Emerging Pandemic Threats programme, to detect potential pandemic illness, there is little financial promise for major pharmaceutical companies to invest in vaccines or drugs for these potential threats until they are a threat to countries that have consumers who can afford them [10]. Had there been significant Ebola outbreaks in affluent nations rather than in Sub-Saharan Africa in the past few decades, we would likely have an arsenal of medications in stock today. While pharmaceutical companies continue to profit from sales of non-essential medicines, and neglect investments in medicines that are needed mainly by the poor, the global community ends up paying as result. Current estimates by the World Bank put the cost of the Ebola outbreak at upwards of US$ 32.6 billion by the end of 2015 – vastly more than what it would have cost to develop effective therapies to stop the epidemic in its tracks [11].

Ultimately, the approach to controlling developing pandemic diseases is multifold. Strengthening health systems, as discussed by Boozary et al., will be important for controlling the spread of disease [12]. However, without access to medications, strong health systems can only do so much to prevent transmission and provide effective care. To cure patients and suppress further transmission, an effective complement to the current pharmaceutical drug development system is urgently needed (**Table 1**).

**Table 1:** Mechanisms to incentivize drug development for Ebola and other diseases of the poor

Mechanism	Pros	Cons	Applied to Ebola
United States Orphan Drug Act of 1983	- Provides up to 50% R&D tax credit for research into “orphan disease”* therapies - Provides 7 year patent exclusivity from time of FDA approval - Fast track approval of drugs to avoid delays	- Does not control pricing, meaning drugs may remain prohibitively costly - Does not incentivize drug development for illnesses affecting people outside the USA	- While pharmaceutical companies are incentivized to produce Ebola medications under this act, they are in no way required to provide these at a reasonable cost to those in West Africa or elsewhere
Priority Review Vouchers	- Allows up to one year advancement in patent approval for blockbuster drug when separate patent is concurrently filed for neglected tropical disease† therapy - No increase in cost to tax payers - Vouchers can be sold to other companies for cash or royalties	- Does not control pricing, meaning drugs may remain prohibitively costly - Low use as of 2014 – only 4 vouchers since the program’s inception in 2007	- While pharmaceutical companies are incentivized to produce Ebola medications under this system, they are in no way required to provide these at a reasonable cost to those in West Africa or elsewhere
Advanced Market Commitments (AMC)	- Governments and other agencies provide subsidies for a fixed number of vaccines sold at lower cost in low-income regions	- Limited funding pool that must be reassessed for each development with no guaranteed source of renewal - No incentive to ensure vaccines reach those in need	- If various international entities and the USA government can supply sufficient funding, an AMC could help produce vaccines for global distribution. However, governments alone would be responsible to deliver vaccines to those in need.
Compulsory Licensing	- Ensures that existing patented drugs are available at an affordable price by allowing domestic generic manufacturing before patent period expires	- Does not incentivize drug companies to research and develop medications for illnesses that affect the poor, even if they cause great morbidity and mortality	- Because Ebola has threatened those in the USA and other wealthy nations, medications are now in mass development. If companies keep drug prices prohibitively high, governments can technically issue compulsory licenses to overcome this.
Health Impact Fund	- Focuses R&D on illnesses causing the most morbidity and mortality - Ensures affordable drugs as sales must occur at cost of production - Creates incentive for companies to facilitate drug delivery and ensure positive health outcomes - Allows for competition in an untapped market while not affecting the current patent system	- Wealthy countries would contribute up to 0.03% of their GNI	- Pharmaceutical companies would be incentivized to produce medicines whether Ebola crossed borders or not because of the potential global health impact at stake. They would also be incentivized to aid governments in ensuring that medications reached those in need to improve health outcomes.

R&D – Research & Development, FDA – Food and Drug Administration, AMC – Advance Market Commitment, GNI – Gross National Income

*Orphan diseases are those classified as affecting fewer than 200 000 people in the USA.

†A full list of neglected tropical diseases by the World Health Organization: http://www.who.int/neglected_diseases/diseases/en/.

As described in detail in *The Lancet* by Banerjee et al., the Health Impact Fund (HIF) can play this role and help overcome the current inefficiencies and inequities of the patent system [13]. The HIF would give pharmaceutical innovators the option of registering any new medicine, thereby agreeing to provide it at cost anywhere it is needed. In exchange, the firm is rewarded based on the drug’s actual health impact, in essence its success in reducing morbidity and mortality. The HIF would pay out a fixed amount of money each year, divided among the registered medicines according to their respective health impact. The HIF would be most attractive for products that are expected to have a large global health impact but relatively low profitability under conventional monopoly pricing. If most countries agreed to contribute around 0.01% of their GNI, the HIF could get started with annual reward pools of US$ 6 billion.

Ebola is no isolated case. Several hundred new infectious diseases have emerged in the last century, mostly in low-income regions, and under present rules global market forces have proven insufficient to promote innovation. By rewarding health impact regardless of the patient’s socioeconomic status, the HIF would provide strong incentives to study such diseases, to develop remedies against them and to promote optimal use of treatments even in the poorest regions [14]. The HIF would answer a moral imperative—to respect and protect the health and lives of the poor—as well as a prudential one—to be smart and proactive in our perennial battle against disease.
